# Estimating intrafraction tumor motion during fiducial-based liver stereotactic radiotherapy via an iterative closest point (ICP) algorithm

**DOI:** 10.1186/s13014-019-1401-2

**Published:** 2019-10-29

**Authors:** Wu-zhou Li, Zhi-wen Liang, Yi Cao, Ting-ting Cao, Hong Quan, Zhi-yong Yang, Qin Li, Zhi-tao Dai

**Affiliations:** 1 School of Physics and Technology, Wuhan University, Wuhan, 430022 China; 2 Cancer Center, Union Hospital, Tongji Medical College, Huazhong University of Science and Technology, Wuhan, 430022 China; 3 Department of Radiation Oncology, Cancer Hospital Chinese Academy of Medical Sciences, Shenzhen Center, Shenzhen, 518100 China

**Keywords:** CyberKnife, Fiducial tracking, SBRT, ICP algorithm, Tumor movement

## Abstract

**Background:**

Tumor motion may compromise the accuracy of liver stereotactic radiotherapy. In order to carry out a precise planning, estimating liver tumor motion during radiotherapy has received a lot of attention. Previous approach may have difficult to deal with image data corrupted by noise. The iterative closest point (ICP) algorithm is widely used for estimating the rigid registration of three-dimensional point sets when these data were dense or corrupted. In the light of this, our study estimated the three-dimensional (3D) rigid motion of liver tumors during stereotactic liver radiotherapy using reconstructed 3D coordinates of fiducials based on the ICP algorithm.

**Methods:**

Four hundred ninety-five pairs of orthogonal kilovoltage (KV) images from the CyberKnife stereo imaging system for 12 patients were used in this study. For each pair of images, the 3D coordinates of fiducial markers inside the liver were calculated via geometric derivations. The 3D coordinates were used to calculate the real-time translational and rotational motion of liver tumors around three axes via an ICP algorithm. The residual error was also investigated both with and without rotational correction.

**Results:**

The translational shifts of liver tumors in left-right (LR), anterior-posterior (AP),and superior-inferior (SI) directions were 2.92 ± 1.98 mm, 5.54 ± 3.12 mm, and 16.22 ± 5.86 mm, respectively; the rotational angles in left-right (LR), anterior-posterior (AP), and superior-inferior (SI) directions were 3.95° ± 3.08°, 4.93° ± 2.90°, and 4.09° ± 1.99°, respectively. Rotational correction decreased 3D fiducial displacement from 1.19 ± 0.35 mm to 0.65 ± 0.24 mm (*P*<0.001).

**Conclusions:**

The maximum translational movement occurred in the SI direction. Rotational correction decreased fiducial displacements and increased tumor tracking accuracy.

## Introduction

Traditional radiotherapy can prolong survival for patients with resectable liver cancers [1] but it offers limited efficacy for the treatment of unresectable primary and metastatic liver cancers mainly due to the low whole liver tolerance to radiotherapy [2, 3]. Stereotactic body radiation therapy (SBRT), which is an accurate external beam irradiation method to deliver conformal high doses in a few fractions, has been proven to be an effective treatment modality for liver cancers with an elevated rate of local control [3–8].

However, target motion may compromise the accuracy of liver stereotactic radiotherapy. It was reported that liver motions of up to 25 mm and 55 mm were observed under normal respiration and deep-breathing, respectively [9–11]. The effect of organ motion on dose has also been investigated [12–14]. According to Velec et al. [14], 70% patients involved in their study treated with liver stereotactic radiotherapy had accumulated dose deviations relative to the planned static prescription dose > 5%, ranging from − 15 to 5% in tumors and 42 to 8% in normal tissues. Management of intrafraction motion is crucial to ensure successful liver SBRT so that nearby healthy tissues and critical organs can be spared. Thus, liver tumor translation, rotation and deformation should be considered in both planning and treatment. Many studies have quantified the rigid and non-rigid motions of liver tumors using 4D computed tomography (4DCT) and/or cone beam computed tomography (CBCT) [14–18]. Yet liver tumors are difficult to visualize on X-ray images due to their low contrast against soft tissues around.

An effective solution to this problem is the use of implanted fiducial markers as surrogates of liver tumors [19–21]. Xu et al. [22] proposed a geometric solution to reconstruct the 3D locations of the fiducials and quantified the rigid motion of liver via Least-squares fitting algorithm [23].This is a closed form solution based on singular value decomposition (SVD) of a 3 × 3 matrix derived from two point sets. According to Murphy et al. [24], the basic SVD solution is ambiguous in differentiating reflections from rotations especially for the case of only three fiducials (which means point sets are coplanar.) A reflection is a mapping from a Euclidean space to itself that is an isometry with a hyperplane as a set of fixed points. The matrix of a reflection is orthogonal with determinant − 1. In our study, the basic SVD solution yields 175 reflections in 360 trials involving three fiducials. Thus, the basic SVD method must be carefully implemented in the appropriate situation to avoid failures caused by singularities.

According to Euler’s rotation theorem, any rotation in three dimensional space can be represented as a combination of a unit vector $\hat{\boldsymbol{e}}$ (called the Euler axis) indicating the direction of an axis of rotation, and an angle θ describing the magnitude of the rotation about the axis. The quaternions, firstly described by W.R.Hamilton [25] in 1843, give a simple way to encode this axis–angle representation in four numbers. A quaternion representation of rotation can be written as $\hat{\boldsymbol{q}}={q}_i\boldsymbol{i}+{q}_j\boldsymbol{j}+{q}_k\boldsymbol{k}+{q}_r={\left[{q}_i\kern0.5em {q}_j\kern0.5em {q}_k\kern0.5em {q}_r\right]}^T$. In terms of the Euler axis $\hat{\boldsymbol{e}}={\left[{e}_x\kern0.5em {e}_y\kern0.5em {e}_z\ \right]}^T$ and angle θ, the four components of this quaternion are expressed as follows:

1 $$ {\displaystyle \begin{array}{l}{q}_i={e}_x\sin \theta /2\\ {}{q}_j={e}_y\sin \theta /2\\ {}{q}_k={e}_z\sin \theta /2\\ {}{q}_r=\cos \theta /2.\end{array}} $$

The quaternion-based algorithm doesn’t involve with singular value decomposition, which is preferred for our purpose since reflections are not desired. The iterative closest point (ICP) algorithm is a robust and fast algorithm which has been demonstrated useful in estimating real-time prostate motion [26]. In this study, we aim to estimate the intrafractional rigid motion of liver tumor based on real-time KV X-ray images acquired by CyberKnife stereo imaging system via a quaternion-based ICP algorithm.

## Material and methods

This study was approved by the Institutional Review Board at the Tongji Medical College of Huazhong University of Science and Technology. All methods were carried out in accordance with the relevant guidelines and regulations.

### Patients and data acquisition

Twelve patients previously treated for liver cancer with CyberKnife robotic radiotherapy system (Accuray, Inc., Sunnyvale, CA, USA) between 2015 and 2018 were enrolled in this study. Patients and treatment details are summarized in Table 1. Four patients were implanted with four gold fiducials and the remaining eight were implanted with three fiducial markers near the tumor under computed tomography (CT) guidance. Fiducial markers are cylindrical gold seeds with a length of about 3–6 mm and a diameter of 0.7–1.2 mm. The distance between any two fiducials was greater than 2 cm; and the angle formed by any three fiducials was greater than 15° to avoid overlapping and collinear effects. These implantation procedures were completed about 1 week before planning CT scan to allow a sufficient time interval for fiducial stabilization. CyberKnife Synchrony fiducial tracking method was used during the whole treatment without breathing control.

**Table 1:** Patient characteristics and main treatment details

	Mean ± SD
Age (yr)	58 ± 12
Volume (cm^3^)	5.6 ± 0.6
Prescribed dose (Gy)	44.8 ± 4.5
Dose per fraction (Gy)	9.1 ± 2.2
Fraction(n)	5.1 ± 1.8
Duration per fraction (min)	38.3 ± 7.4

### Marker segmentation

**Fig. 1:**
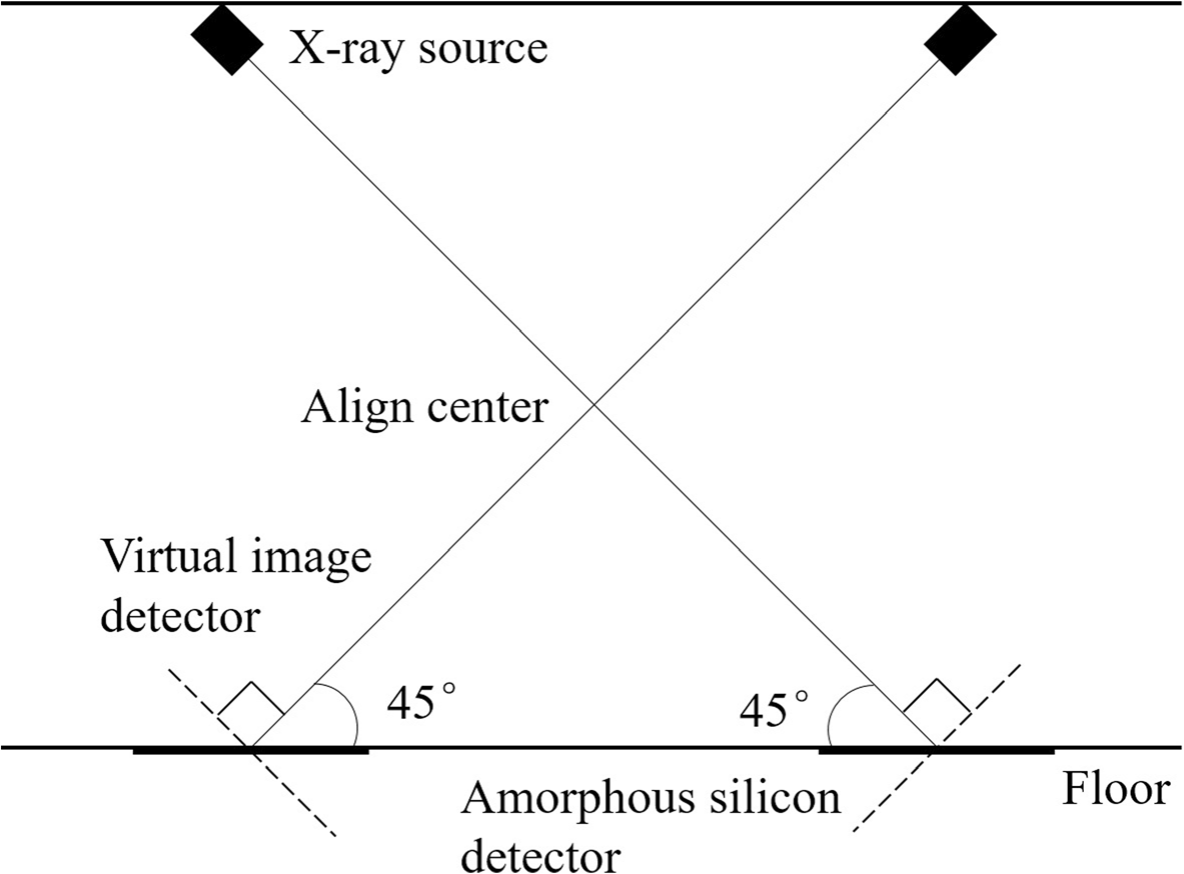
Diagram of the CyberKnife stereo imaging system

**Fig. 2:**
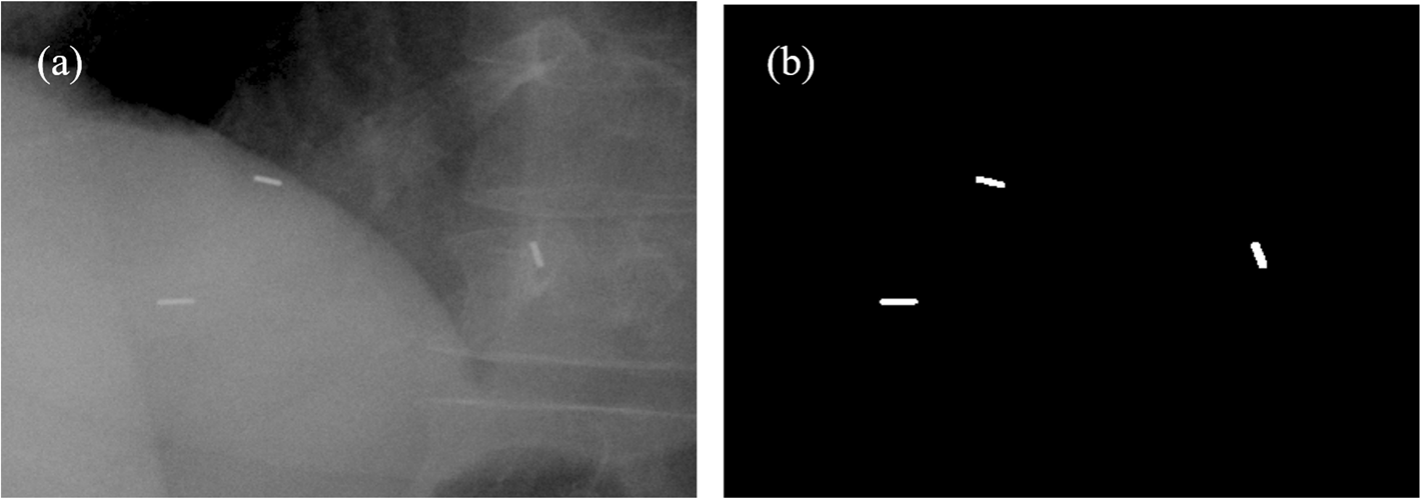
**a** Two-dimensional kV image of three radio opaque fiducial markers. **b** Bitmap obtained after binarization and mean filtering

The CyberKnife image guidance system consists of two orthogonal X-ray sources fixed on the ceiling and two amorphous silicon panel detectors mounted on both sides of the treatment couch. Figure 1 shows a diagram of the CyberKnife imaging system. The time interval between two adjacent X-ray imaging sessions was 40 s. A total of 495 pairs of KV X-ray images were acquired during the first fraction of the treatment of 12 patients. Each image, a 1024 × 1024 matrix, was converted to a binary image containing the number 0.0 (Black) and 1.0 (white). Figure 2a shows a screenshot of a kV image with three fiducial markers; Fig. 2b shows the image after binarization and mean filtering. The centroid of a fiducial marker was defined at the center point of a ‘white blob’, and the two-dimensional (2D) position coordinates of the centroid were derived from the binary image.

### 3D fiducial reconstruction

**Fig. 3:**
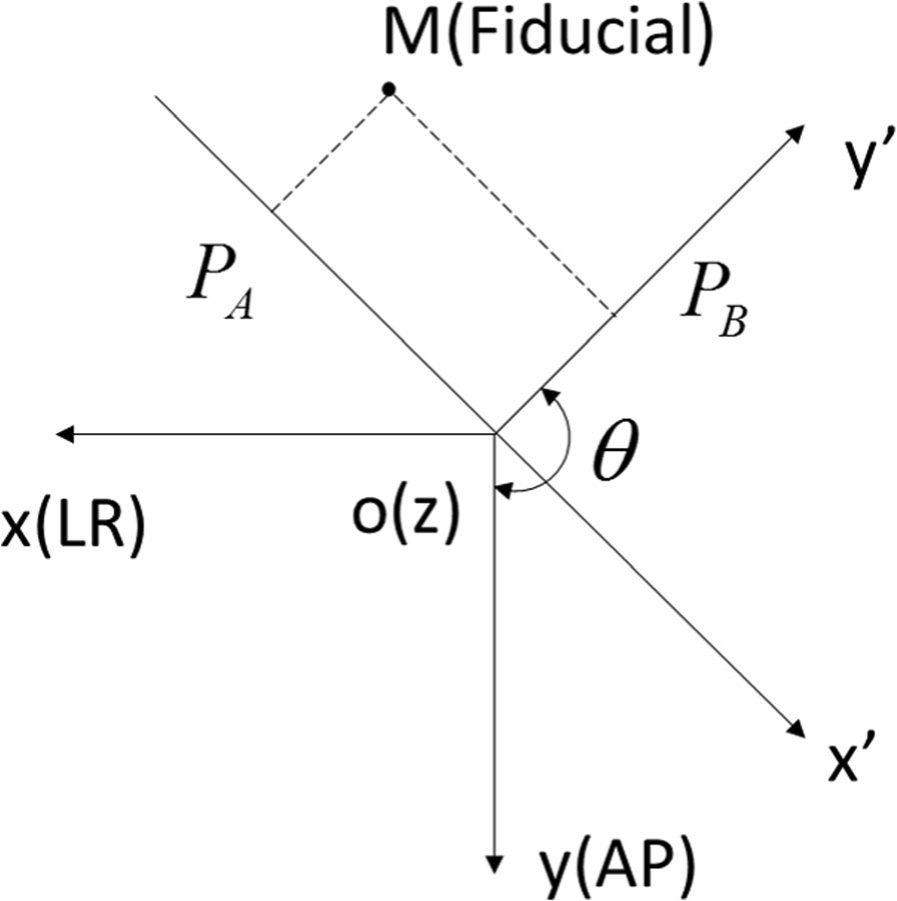
Diagram showing projection and coordinate system rotation. The fiducial marker is at M; *P*_*A*_ and *P*_*B*_ are the two projection points

The fiducial marker is viewed from two orthogonal camera positions, and the 3D coordinates can be reconstructed at the intersection of the back projections towards the source [27]. Parallel rays were assumed for simplicity. A point object (e.g. a fiducial marker) was positioned at the point M (Fig. 3). Point *P*_*A*_ and point *P*_*B*_ are projections of point M on the correspondent image, of which two-dimensional coordinates on the respective image are known. Let the coordinates of the projection point *P*_*A*_ be called (*u*_*a*_, *v*_*a*_), and for point *P*_*B*_ be called (*u*_*b*_, *v*_*b*_). The coordinates of point M is denoted by (*α*^′^, *β*^′^, *γ*^′^) in the image coordinate system (*x*^′^*y*^′^*z*^′^) and (*α*, *β*, *γ*) in the patient coordinate system (*xyz*). The coordinate of both projection points along the SI (Superior-Inferior) direction (z-axis) are theoretically equal. Thus, the 3D coordinates of point M can be derived as shown in Eq. 2, after combining geometrical information.

2 $$ \left(\begin{array}{c}x\\ {}y\\ {}z\end{array}\right)=\left(\begin{array}{ccc}\cos \theta & -\sin \theta & 0\\ {}\sin \theta & \cos \theta & 0\\ {}0& 0& 1\end{array}\right)\left(\begin{array}{c}{u}_a\\ {}{u}_b\\ {}\left({v}_a+{v}_b\right)/2\end{array}\right) $$

The reconstruction algorithm was validated using the stereotactic dose verification phantom (SDVP; Standard Imaging, Inc., Middleton, WI, USA), which was illustrated in Additional file 1. The results show that the mean difference between the reconstructed 3D fiducial coordinates and those recorded in the CyberKnife log file is 0.72 mm.

### Estimation of translational and rotational motions

Assuming two sets of data points for fiducial markers, the set of target data points (denoted as *Y* = {*y*_1_, *y*_2_, …*y*_*n*_}) was translated and rotated to the set of reference data points (denoted as X = {*x*_1_, *x*_2_, …*x*_*n*_}). Due to liver tumor deformation and fiducial marker migration, it is not possible to find a transformation that perfectly maps the two sets of fiducial markers. The aim of the ICP algorithm was to find the rotation matrix *R* and translation vector *T* that minimizes the following objective function:

3 $$ {\sum}^2=\frac{1}{n}\sum \limits_{i=1}^n\left\Vert {x}_i\right.-{\left.\left({Ry}_i+T\right)\right\Vert}^2 $$

**Fig. 4:**
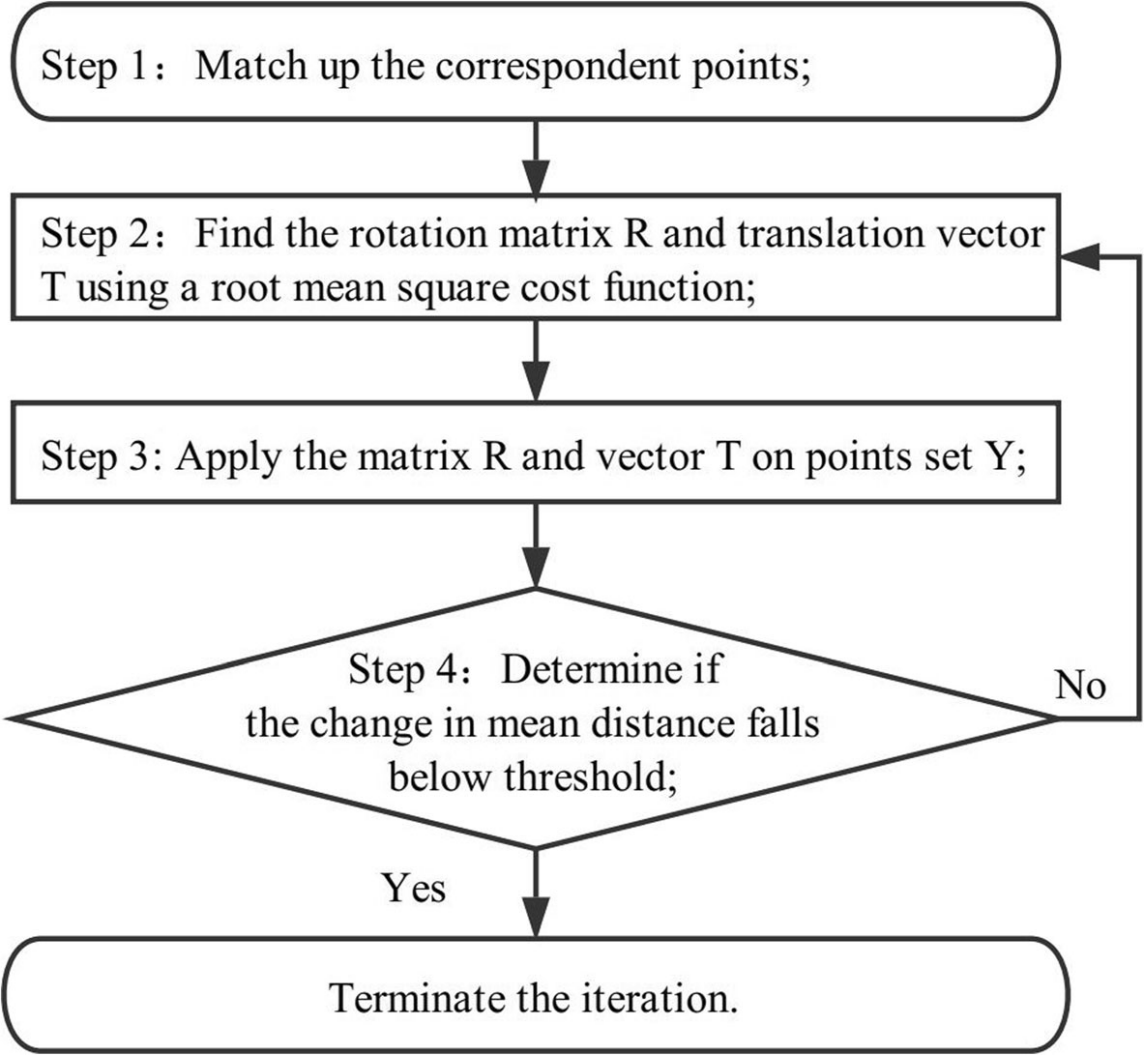
Flow chart of the ICP algorithm implemented in 3D fiducial registration

The flow chart of the ICP algorithm is shown in Fig. 4.

**Step 1**: the ICP algorithm is based on the nearest neighbor decision rule [28] to match up the corresponding points.

**Step 2 and 3**: computed the rotation matrix *R* and translation vector *T* that minimizes the mean square error of the estimated corresponding pairs.

**Step 4**: the threshold was set to 0.001 mm. Typically, it takes no more than 2 or 3 iterations before achieving convergence.

The derivation of the quaternion-based ICP algorithm used in our study is described as follows: Assuming the rotation matrix *R* is denoted by $R=q={\left[{q}_0\kern0.5em {q}_1\kern0.5em {q}_2\kern0.5em {q}_3\right]}^T$.

The centroids of point set *X* and point set *Y* are given by

4 $$ \left\{\begin{array}{c}{\mu}_x=\frac{1}{n}\sum \limits_{i=1}^n{x}_i\\ {}{\mu}_y=\frac{1}{n}\sum \limits_{i=1}^n{y}_i\end{array}\right. $$

and the cross-covariance matrix of sets *Y* and *X* can be written as follows:

5 $$ {\sum}_{yx}=\frac{1}{n}\sum \limits_{i=1}^n\left[\left({y}_i-{\mu}_y\right){\left({x}_i-{\mu}_x\right)}^T\right]=\frac{1}{n}\sum \limits_{i=1}^n{y}_i{x_i}^T-{\mu}_y{\mu_x}^T $$

Construct matrix A:

6 $$ {A}_{ij}={\left({\sum}_{yx}-{\sum}_{yx}^T\right)}_{ij} $$

Matrix A is used to construct the column vector ∆:

7 $$ \Delta ={\left[{A}_{23}\kern0.5em {A}_{31}\kern0.5em {A}_{12}\right]}^T $$

Vector ∆ is applied to yield a symmetric matrix Q:

8 $$ Q=\left[\begin{array}{cc} tr\left({\sum}_{yx}\right)& {\Delta}^T\\ {}\Delta & {\sum}_{yx}+{\sum}_{yx}^T- tr\left({\sum}_{yx}\right){I}_3\end{array}\right] $$

where tr(•) denotes the trace of a matrix, and *I*_3_ is a 3 × 3 identity matrix.

According to the studies of Besl et al. [29] and Horn et al. [30], the unit eigenvector $q={\left[{q}_0\kern0.5em {q}_1\kern0.5em {q}_2\kern0.5em {q}_3\right]}^T$ corresponding to the maximum eigenvalue of the matrix *Q* is regarded as the quaternion that minimizes the objective function (3). According to *q*, the rotation matrix *R* can be written as:

9 $$ R=\left[\begin{array}{ccc}{q}_0^2+{q}_1^2-{q}_2^2-{q}_3^2& 2\left({q}_1{q}_2-{q}_0{q}_3\right)& 2\left({q}_1{q}_3+{q}_0{q}_2\right)\\ {}2\left({q}_1{q}_2+{q}_0{q}_3\right)& {q}_0^2+{q}_2^2-{q}_1^2-{q}_3^2& 2\left({q}_2{q}_3-{q}_0{q}_1\right)\\ {}2\left({q}_1{q}_3-{q}_0{q}_2\right)& 2\left({q}_2{q}_3+{q}_0{q}_1\right)& {q}_0^2+{q}_3^2-{q}_1^2-{q}_2^2\end{array}\right] $$

After R was solved, the translation vector *T* could be derived:

10 $$ T={\mu}_x-R{\mu}_y $$

### Statistical analyses

The fiducials in a pair of orthogonal images near the beginning of each treatment were used as reference points set, and the registration residual errors with translational corrections only and with rigid corrections are also recorded. Statistical analysis was performed using paired sample t-test analysis with SPSS Statistics 20 (IBM, Armonk, USA) software. The null hypothesis is that the true difference between registration errors with translational corrections only and with rigid corrections is zero. *P* value < 0.001 was considered statistically significant at 95% confidence level.

## Results

A total of 495 pairs of kV X-ray images from 12 patients were analyzed in this study. The translational and rotational motion ranges are summarized in Table 2. Translation and rotation are measured as the mean of the maximum range of motion for each case. For all patients, the translational motion ranges in left-right (LR), anterior-posterior (AP), and superior-inferior (SI) directions were 2.92 ± 1.98 mm (∆x), 5.53 ± 3.12 mm (∆y), and 16.22 ± 5.86 mm (∆z), respectively. Translational motion range in 3D space (Δd) can be computed as:

11 $$ \Delta \mathrm{d}=\sqrt{\Delta {\mathrm{x}}^2+\Delta {\mathrm{y}}^2+\Delta {\mathrm{z}}^2} $$

**Table 2:** The mean and standard deviation (SD) of translational and rotational motion ranges of liver tumors in each direction

	∆x (mm)	∆y (mm)	∆z (mm)	∆d (mm)	∆θ_x_(°)	∆θ_y_(°)	∆θ_z_(°)
Mean	2.92	5.54	16.22	11.89	3.95	4.93	4.09
SD	1.98	3.12	5.86	5.11	3.08	2.90	1.99

The translational motion range in 3D space was 11.89 ± 5.11 mm (∆d).

**Fig. 5:**
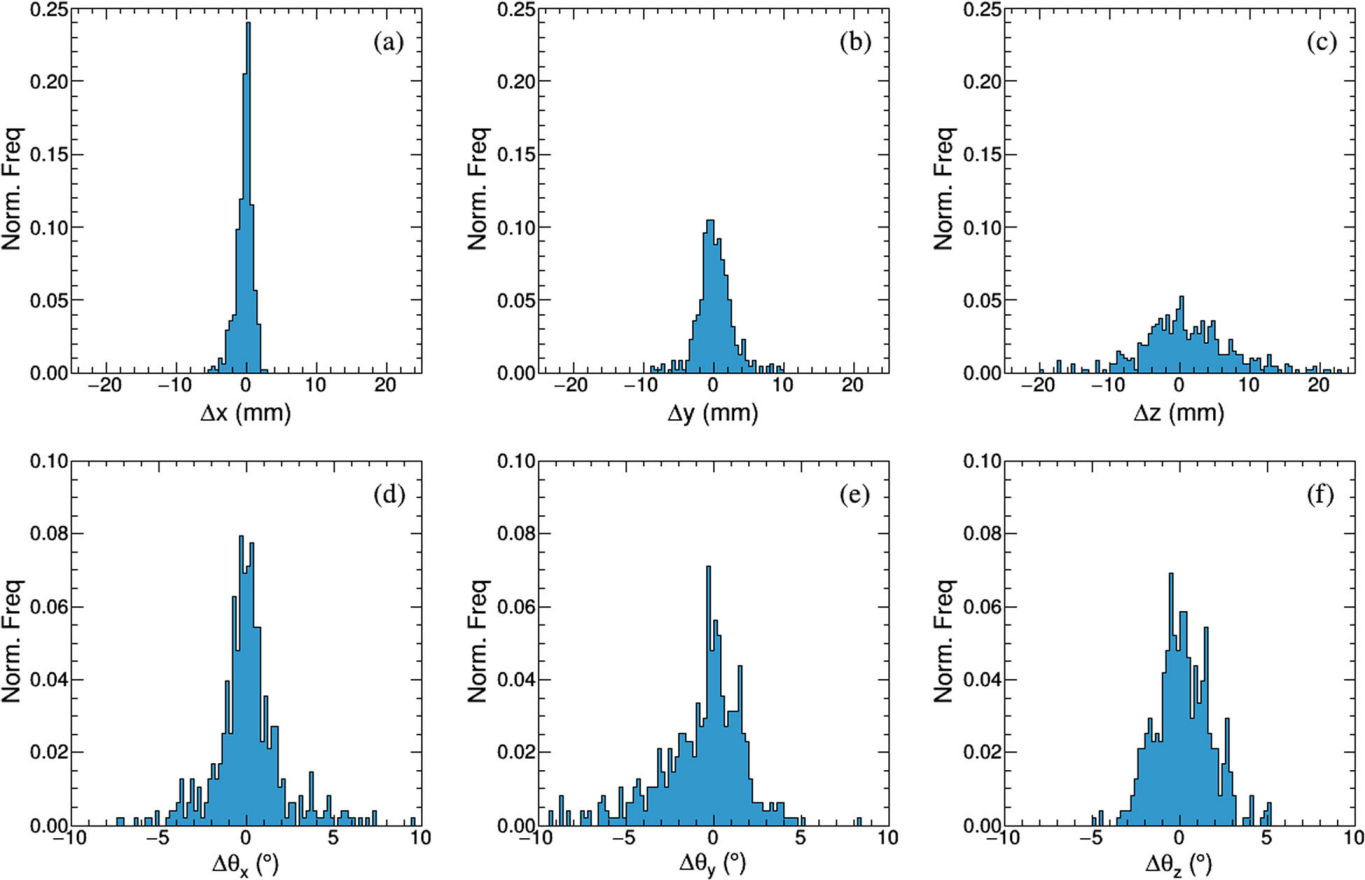
Normalized histogram of translational movements in the (**a**) Left-Right LR (Δx), (**b**) Anterior-Posterior AP (Δy), (**c**) Superior-Inferior SI (Δz) directions respectively. Normalized histogram of rotational movements in the (**d**) Left-Right LR (Δθ_x_), (**e**) Anterior-Posterior AP (Δθ_y_), (**f**) Superior-Inferior SI (Δθ_z_) directions, respectively

**Fig. 6:**
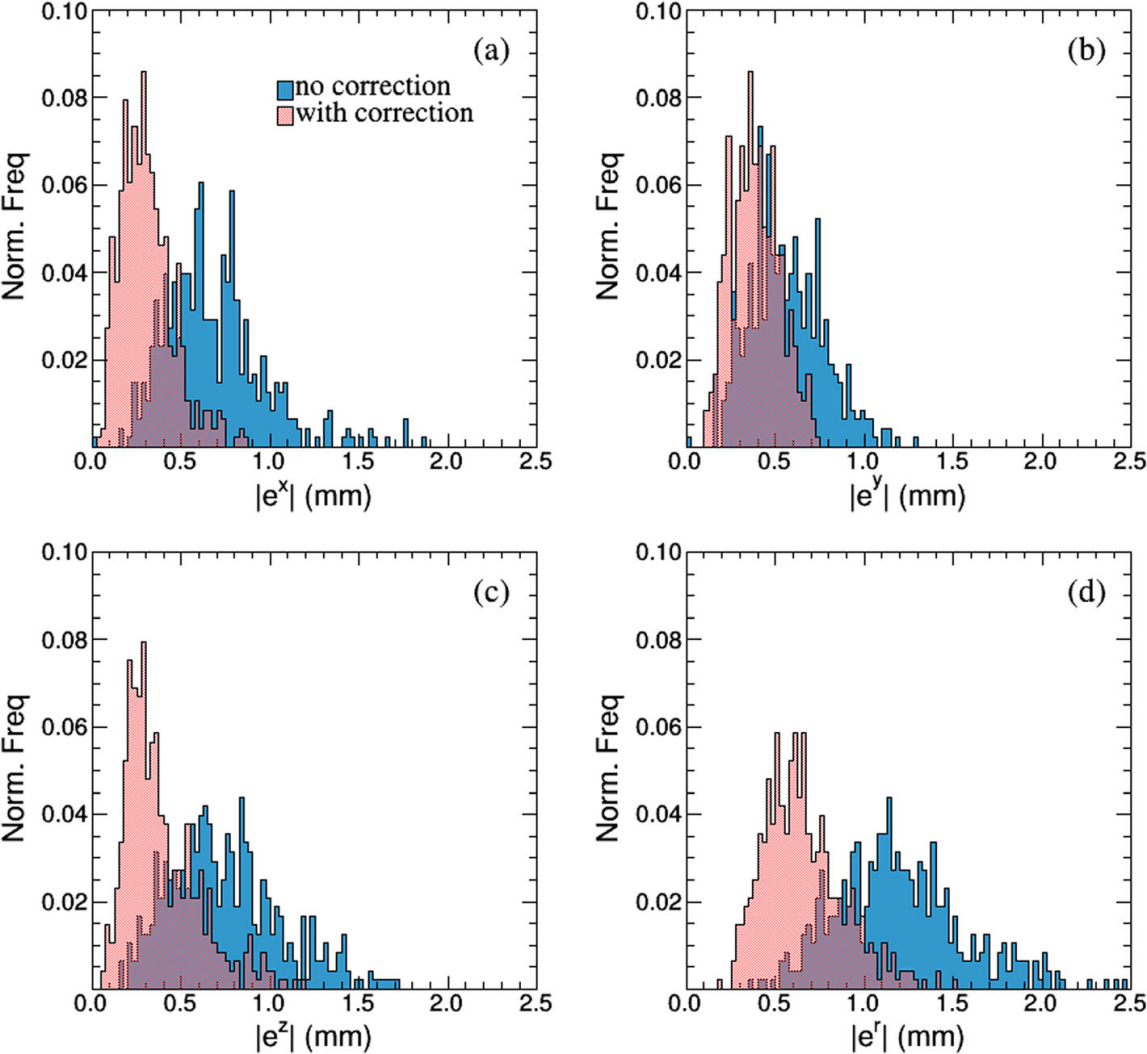
Fiducial registration residual errors in the (**a**) Left-Right LR (|e^x^|), (**b**) Anterior-Posterior AP (|e^y^|), (**c**) Superior-Inferior SI (|e^z^|) directions, respectively. (**d**) Residual errors in 3D space (|e^r^|). The blue area indicates with translational corrections only; the red area indicates with rigid corrections

The rotational angles in LR, AP, and SI directions were 3.95° ± 3.08° (∆θ_x_), 4.93° ± 2.90° (∆θ_y_), and 4.09° ± 1.99° (∆θ_z_), respectively. Figure 5 displays a normalized frequency histogram of intrafractional translational shift and rotational angles of all patients. Large rotation angles exceeding 8° were only observed in two cases. Figure 6 shows the registration residual error between the reference points and the transformed target points with rigid corrections (Fig. 6, red) and with translational corrections only (Fig. 6, blue). The mean ± SD of residual errors with and without rotational correction in each direction are displayed in the Table 3. The difference in each direction was statistically significant with and without rotational correction (*P* < 0.001). Rotational corrections decreased 3D residual error from 1.19 ± 0.35 mm to 0.68 ± 0.24 mm.

**Table 3:** Residual error with and without (w/o) rotational corrections. Statistical analysis was performed using paired sample t-test analysis

	Error w/o rotational correction	Error with rotational correction	*p*-value
	Mean ± SD	Mean ± SD	
|e^x^|(mm)	0.68 ± 0.26	0.31 ± 0.15	< 0.001
|e^y^|(mm)	0.57 ± 0.21	0.39 ± 0.13	< 0.001
|e^z^|(mm)	0.74 ± 0.31	0.39 ± 0.21	< 0.001
|e^d^|(mm)	1.19 ± 0.36	0.65 ± 0.23	< 0.001

## Discussion

In this study, the intrafraction translations and rotations of liver tumor were estimated via the ICP algorithm during CyberKnife-based stereotactic radiotherapy. The maximum translational motion occurred in the SI direction due to breathing motion, and the smallest translation was observed along the LR direction, which agrees with previous studies [15, 18, 20, 21]. Xu et al. [22] obtained kV images from CyberKnife imaging system and reported that the means ± SD of the absolute value of intrafraction translations in liver SBRT was 2.1 ± 2.3 mm (LR), 2.9 ± 2.8 mm (AP), and 6.4 ± 5.5 mm (SI). Higher values were obtained in this study, since the maximum motion range for each patient was used to calculate the mean and standard deviation of both translation and rotation. In addition to translational movements, large rotational motion angles were observed. This suggests that the rigid motion of liver tumor should be paid special attention to for some patients. The residual error with rotational correction was 0.65 ± 0.23 mm, probably due to liver tumor deformation during treatment.

Several ways can be utilized to compute the optimal rigid transformation of two geometric data sets. Matching up the correspondent point one-to-one between the reference image and the test image is necessary to solve this problem. Distance between fiducials should exceed 20 mm to avoid overlapping or mismatching [24]. It is not difficult to associate the target point with the corresponding point in reference image in our case. Once the correspondence is known, the orthogonal transformation that minimizes the residual error in points set registration can be found with both of the closed form and iterative methods. This indicates that true least-squares minimum exists in the solution and the convergence of the iterative algorithm is guaranteed. The closest iterative point (ICP) algorithm is commonly used in medical image registration [31, 32], and it was found to be more general and robust when dealing with corrupted data which is more common in realistic scenarios.

The algorithm for fiducial segmentation in this study might introduce minor errors. Firstly, the resolution of kV images obtained by CyberKnife imaging system was 1024 × 1024. Thus, the centroid of the ‘white blobs’ calculated by the coordinates of the pixel block may deviate from the actual centroid of the gold fiducial markers. Secondly, the process of binarization could also introduce minor errors to the calculation of the fiducial centroids. Automatic solutions to segment radiopaque fiducial markers from CBCT scans have been proposed previously [27, 33, 34]. Mao et al. [33] presented a pattern matching algorithm using matched filters and template matching for detecting fiducials specifically designed to work with both kV and MV image data. However, this algorithm might be less applicable in this study because the projections of fiducials change with projection angle caused by liver deformation. Other solutions using CBCT projections with large angular separation and good marker contrast on a uniform background might also be not suitable in this study. Considering that the image data used in this study contained considerable noise caused by the bone structure, false signals are almost inevitable with these methods. Fiducial trajectory estimation is important in tumor motion management such as tumor tracking. A robust and reliable automatic segmentation method used in CyberKnife-based kV X-ray images needs to be developed.

## Conclusion

Tumor motion management is necessary to improve the accuracy of SBRT. The rotational and translational motions of liver tumors during SBRT were estimated based on the CyberKnife system via the ICP algorithm. The residual error after registration decreased significantly with rotational correction. The results of this study can be used in motion management and planning target volume (PTV) margin determination for both liver SBRT and conventional radiotherapy.

## Supplementary information

**Additional file 1.** The detailed information about SDVP.

## Data Availability

All data included in this study are available upon request by contact with the corresponding author.

## Abbreviations

3D: Three-dimensional
4DCT: 4D computed tomography
AP: Anterior-posterior
CBCT: Cone beam computed tomography
CT: Computed tomography
ICP: Iterative closest point
KV: Kilovoltage
LR: Left-right
MV: Megavoltage
PTV: Planning target volume
SBRT: Stereotactic body radiation therapy
SDVP: Stereotactic dose verification phantom
SI: Superior-inferior
SVD: Singular value decomposition
