# Supplementary material for: Estimating intrafraction tumor motion during fiducial-based liver stereotactic radiotherapy via an iterative closest point (ICP) algorithm
Source: Radiat Oncol. 2019 Oct 29;14:185. doi: 10.1186/s13014-019-1401-2 (PMC6820939; doi:10.1186/s13014-019-1401-2)
Supplement: Supplementary file 1 — Additional file 1. The detailed information about SDVP. [file 13014_2019_1401_MOESM1_ESM.docx]

**Supplementary material**

The supplementary material contains three parts:

1. The code for the ICP method.
2. The code for the basic SVD method.
3. The information about stereotactic dose verification phantom (SVDP).
4. Part 1: The code for the ICP method.

Step 1: Read the KV images and perform image processing. Calculate the fiducial coordinate on the image.

clear;

clc;

I = imread('File path\file name.png');

%imshow(I);

I_filtered = filter2(fspecial('average',3),I)/255;

I_bw = im2bw(I);

%imshow(I_bw);

[row, col] = find( I_bw ~= 0 );

a_max = max(row);

b_max = max(col);

a_min = min(row);

b_min = min(col);

Step 2: Read the fiducial coordinates and reconstruct the fiducial in patient system.

And store the data.

function DataMatrix = ReadFdc(NameOfXlsx, NameOfFdc, Writexls)

A = xlsread(NameOfXlsx,NameOfFdc);

[Column, Row] = size(A);

halfColumn = Column/2;

A_1 = zeros(Column,2);

A_1(:,1)=0.5*(A(:,1)+A(:,3));

A_1(:,2)=0.5*(A(:,2)+A(:,4));

B=zeros(halfColumn,3);

for i=1:halfColumn

B(i,1)=(A_1(2*i-1,1)-1024)*0.25/2^0.5; B(i,2)=A_1(2*i,1)*0.25/2^0.5; B(i,3)=0.5*0.25*(A_1(2*i-1,2)+A_1(2*i,2));

end

theta = pi*3/4;

Tran = [cos(theta) -sin(theta) 0

sin(theta) cos(theta) 0

0 0 1];

C_1=zeros(3,halfColumn);

%DataMatrix = zeros(37,3);

DataMatrix = zeros(halfColumn,3);

C_1=Tran*B.';

DataMatrix = C_1.';

xlswrite( Writexls, DataMatrix);

end

Step 3: Plot the reconstructed fiducials.

clear;

clc;

close all;

DataMatrix_1 = ReadFdc('f8.xlsx', 'fiducial 1', 'C_fiducial_1.xls');

DataMatrix_2 = ReadFdc('f8.xlsx', 'fiducial 2', 'C_fiducial_2.xls');

DataMatrix_3 = ReadFdc('f8.xlsx', 'fiducial 3', 'C_fiducial_3.xls');

%DataMatrix_4 = ReadFdc('f.xlsx', 'fiducial 4', 'C_fiducial_4.xls');

hold on %Retain current plot when adding new plots

grid on %Display axes grid lines

plot3(DataMatrix_1(:,1),DataMatrix_1(:,2),DataMatrix_1(:,3),'bd')

plot3(DataMatrix_2(:,1),DataMatrix_2(:,2),DataMatrix_2(:,3),'rd')

plot3(DataMatrix_3(:,1),DataMatrix_3(:,2),DataMatrix_3(:,3),'gd')

Step 4: Use the ICP method.

Put in two data sets: data_g and data_p

Put out: The data sets after rotation, the rotation matrix and residual error.

function [ data_g, data_p, err, data_pp, R] = icp_process( data_g, data_p )

[k1, n] = size(data_g);

[k2, m] = size(data_p);

data_p1 = zeros(k2, 3);

data_pp = zeros(k1, 3);

distance = zeros(k1, 1);

error = zeros(k1, 1);

data_g = normal_gravity(data_g);

data_p = normal_gravity(data_p);

% Nearest neighbor rule.

for i = 1:k1

data_p1(:, 1) = data_p(:, 1) - data_g(i, 1);

data_p1(:, 2) = data_p(:, 2) - data_g(i, 2);

data_p1(:, 3) = data_p(:, 3) - data_g(i, 3);

distance = data_p1(:, 1).^2 + data_p1(:, 2).^2 + data_p1(:, 3).^2;

[min_dis, min_index] = min(distance);

data_pp(i, :) = data_p(min_index, :);

error(i) = min_dis;

end

V = (data_g' * data_pp) ./ k1;

matrix_Q = [V(1,1)+V(2,2)+V(3,3),V(2,3)-V(3,2),V(3,1)-V(1,3),V(1,2)-V(2,1);

V(2,3)-V(3,2),V(1,1)-V(2,2)-V(3,3),V(1,2)+V(2,1),V(1,3)+V(3,1);

V(3,1)-V(1,3),V(1,2)+V(2,1),V(2,2)-V(1,1)-V(3,3),V(2,3)+V(3,2);

V(1,2)-V(2,1),V(1,3)+V(3,1),V(2,3)+V(3,2),V(3,3)-V(1,1)-V(2,2)];

[V2, D2] = eig(matrix_Q);

lambdas = [D2(1, 1), D2(2, 2), D2(3, 3), D2(4, 4)];

[lambda, ind] = max(lambdas);

Q = V2(:, ind);

% Calculate the rotation matrix based on quaternions.

R=[Q(1,1)^2+Q(2,1)^2-Q(3,1)^2-Q(4,1)^2, 2*(Q(2,1)*Q(3,1)-Q(1,1)*Q(4,1)), 2*(Q(2,1)*Q(4,1)+Q(1,1)*Q(3,1));

2*(Q(2,1)*Q(3,1)+Q(1,1)*Q(4,1)), Q(1,1)^2-Q(2,1)^2+Q(3,1)^2-Q(4,1)^2, 2*(Q(3,1)*Q(4,1)-Q(1,1)*Q(2,1));

2*(Q(2,1)*Q(4,1)-Q(1,1)*Q(3,1)), 2*(Q(3,1)*Q(4,1)+Q(1,1)*Q(2,1)), Q(1,1)^2-Q(2,1)^2-Q(3,1)^2-Q(4,1)^2;

];

data_p = data_p * R;

data_pp = data_pp * R;

data_p = normal_gravity(data_p);

data_pp = normal_gravity(data_pp);

err = mean(error);

%displacement = error;

end

Step 5: Find the rotation matrix that minimize the object function by iterative method.

function [R,error, convergeSet, withoutRoationDisplacement, withRoationDisplacement ] = rotation(p1,P)

data_g = p1;

data_p = P;

convergeSet = zeros(1,0);

[m,n] = size(data_g);

temp_withoutRoationDisplacement = normal_gravity(data_g) - normal_gravity(data_p);

for i = 1:n

withoutRoationDisplacement(i) = sum(abs(temp_withoutRoationDisplacement(:,i)))/m;

end

%plot_3d_2(data_g, data_p, -90);

[ data_g, data_p, error, data_pp, R ] = icp_process( data_g, data_p );

log_info(strcat('Number of iteration：1，Current error：', num2str(error)));

log_info('Current rotation matrix：');

disp(R);

convergeSet(1) = error;

cnt = 1;

last_error = 0;

last_R = R;

%%Two ways to achieve convergence.

for cnt = 2:10 %Fixed number iteration

% while abs(error - last_error) > 0.001 %Set a threshold to achieve convergence.

% cnt = cnt + 1;

last_error = error;

last_R = R;

[ data_g, data_p, error, data_pp, R] = icp_process( data_g, data_p );

convergeSet(cnt) = error;

R = last_R * R;

log_info(strcat('Number of Iteration：', num2str(cnt), '，Error：', num2str(error)));

log_info('The current rotation matrix is：');

disp(R);

end

temp_withRoationDisplacement = data_g - data_pp;

for i = 1:n

withRoationDisplacement(i) = sum(abs(temp_withRoationDisplacement(:,i)))/m;

end

%plot_3d_2(data_g, data_p, -90);

end

Step 6. Calculate the translation matrix.

function [T] = translation()

clear;

clc;

Fiducial_1 = xlsread('C_fiducial_1');

Fiducial_2 = xlsread('C_fiducial_2');

Fiducial_3 = xlsread('C_fiducial_3');

[m,n] = size(Fiducial_1);

C = 1/3*(Fiducial_1+Fiducial_2+Fiducial_3);

T = zeros(m,3);

for i=1:m

T(i,:) = C(i,:)-C(1,:);

end

end

Step 7: The main function. Plot the histogram.

clear;

clc;

serialNum = importdata('Num.txt');

serialNum = num2str(serialNum);

xlsName = strcat('data',serialNum)

Fiducial_1 = xlsread('C_fiducial_1');

Fiducial_2 = xlsread('C_fiducial_2');

Fiducial_3 = xlsread('C_fiducial_3');

existance4 = exist('C_fiducial_4','file')

if (existance4)

Fiducial_4 = xlsread('C_fiducial_4');

p1(4,:) = Fiducial_4(1,:);

end

p1(1,:) = Fiducial_1(1,:);

p1(2,:) = Fiducial_2(1,:);

p1(3,:) = Fiducial_3(1,:);

[m,n] = size(Fiducial_1);

ROTATION = zeros(3,3,m-1);

ERROR = zeros(m-1,1);

TRANSLATION =zeros(m-1,3);

ANGLE =zeros(m-1,3);

plotCpvergeSetNum = 10;

convergeSet = zeros(1,10);

temp_convergeSet = zeros(1,10);

withoutRotationDisplacement_matrix = zeros(m-1,3);

withRotationDisplacement_matrix = zeros(m-1,3);

Displacement_matrix = zeros(m-1,6);

for i = 2:m

P(1,:) = Fiducial_1(i,:);

P(2,:) = Fiducial_2(i,:);

P(3,:) = Fiducial_3(i,:);

if (existance4)

P(4,:) = Fiducial_4(i,:);

end

[R,error, temp_convergeSet, withoutRotationDisplacement, withRotationDisplacement] = rotation(p1,P);

if(i == plotCpvergeSetNum )

convergeSet = temp_convergeSet;

end

withoutRotationDisplacement_matrix(i-1,:) = withoutRotationDisplacement;

withRotationDisplacement_matrix(i-1,:) = withRotationDisplacement;

Displacement_matrix(i-1, 1:3) = withoutRotationDisplacement;

Displacement_matrix(i-1, 4:6) = withRotationDisplacement;

ROTATION(:,:,i-1) = R;

ERROR(i-1,:)= error;

ANGLE(i-1,:)= rotationMatrix2eulerAngles(R);

end

PlotX = 1:10;

figure(1)

plot(PlotX, convergeSet,'bo')

xlswrite(strcat('E:\fiducial\data\',serialNum,'.xlsx'),Displacement_matrix)

figure(2)

xyz = 1;

maximumNum = max(withoutRotationDisplacement_matrix(:,xyz));

if (max(withRotationDisplacement_matrix(:,xyz)) > maximumNum)

maximumNum = max(withRotationDisplacement_matrix(:,xyz));

end

h = histogram(withoutRotationDisplacement_matrix(:,xyz), [0:0.1:maximumNum * 1.2] );

set(h,'FaceColor','r');

hold on

h = histogram(withRotationDisplacement_matrix(:,xyz), [0:0.1:maximumNum * 1.2]);

set(h,'FaceColor','k');

if (existance4)

C = 1/4*(Fiducial_1+Fiducial_2+Fiducial_3 + Fiducial_4);

else

C = 1/3*(Fiducial_1+Fiducial_2+Fiducial_3);

end

for j=2:m

TRANSLATION(j-1,:) =( C(j,:)-C(1,:));

end

Other functions:

%%Put in: The original point sets and rotation angle

Put out: The new point sets after it has been rotated clockwise around the x axis

function new_data = rotate( data, theta )

theta = - theta * pi / 180;

matrix_rotate = [1, 0, 0, 0;

0, cos(theta), sin(theta), 0;

0, -sin(theta), cos(theta), 0;

0, 0, 0, 1];

rows = size(data, 1);

row_ones = ones(rows, 1);

new_data = [data, row_ones] * matrix_rotate;

new_data = new_data(:, 1:3);

end

%% Display information in the window

function log_info( input_str )

time_str = datestr(now, 31);

info_str = strcat('[', time_str, '] ', input_str);

disp(info_str);

end

%%Change rotation matrix to Euler angles.

function eulerAngles = rotationMatrix2eulerAngles(R)

if abs(R(3,1)) ~= 1

theta1 = -asin(R(3,1));

theta2 = pi - theta1;

psi1 = atan2(R(3,2)/cos(theta1), R(3,3)/cos(theta1));

psi2 = atan2(R(3,2)/cos(theta2), R(3,3)/cos(theta2));

pfi1 = atan2(R(2,1)/cos(theta1), R(1,1)/cos(theta1));

pfi2 = atan2(R(2,1)/cos(theta2), R(1,1)/cos(theta2));

theta = theta1; % could be any one of the two

psi = psi1;

pfi = pfi1;

else

phi = 0;

delta = atan2(R(1,2), R(1,3));

if R(3,1) == -1

theta = pi/2;

psi = phi + delta;

else

theta = -pi/2;

psi = -phi + delta;

end

end

eulerAngles = [psi*180/pi theta*180/pi pfi*180/pi]; %for degree;

function ret = normal_gravity( data )

[m, n] = size(data);

data_mean = mean(data);

ret = data - ones(m, 1) * data_mean;

end

function plot_3d( data, theta )

data = rotate(data, theta);

x = data(:, 1);

y = data(:, 2);

z = data(:, 3);

% plot3(x, y, z, 'k');

figure();

scatter3(x, y, z, 'k');

end

function d = distance(a,b)

d= sqrt((a-b)*(a-b).');

end

function save_3d_data( path, data )

f = fopen(path, 'w');

[m, n] = size(data);

for i = 1:m

for j = 1:n

if j == n

fprintf(f, '%g\r\n', data(i, j));

else

fprintf(f, '%g ', data(i, j));

end

end

end

fclose(f);

end

function plot_3d_2( data1, data2, theta )

data1 = rotate(data1, theta);

x1 = data1(:, 1);

y1 = data1(:, 2);

z1 = data1(:, 3);

data2 = rotate(data2, theta);

x2 = data2(:, 1);

y2 = data2(:, 2);

z2 = data2(:, 3);

figure();

scatter3(x1, y1, z1, 'b');

hold on;

scatter3(x2, y2, z2, 'r');

hold off;

end

1. Part 2: The SVD method.

The code for the image reading and processing section has been written in the previous section. The matrix it yields is denoted by X.

If det(X) = 1, X is a rotation matrix;

If det(X) = -1, X is a reflection matrix.

The code for calculating the rotation matrix by the SVD algorithm is as follows:

clear;

clc;

Fiducial_1 = xlsread('File path\C_fiducial_1');

Fiducial_2 = xlsread('File path\C_fiducial_2');

Fiducial_3 = xlsread('File path\C_fiducial_3');

Fiducial_4 = xlsread('File path\C_fiducial_4');

p_1 = Fiducial_1(1,:); p_2 = Fiducial_2(1,:);

p_3 = Fiducial_3(1,:);

p_4 = Fiducial_4(1,:);

P_centroid =1/3*( p_1 + p_2 +p_3+p_4);

Q_1 = p_1 - P_centroid;

Q_2 = p_2 - P_centroid;

Q_3 = p_3 - P_centroid;

Q_4 = p_4 - P_centroid;

q_1 = Q_1';

q_2 = Q_2';

q_3 = Q_3';

q_4 = Q_4';

[m,n]= size(Fiducial_1);

H = zeros(3,3);

X = zeros(3,3);

R = zeros(3,3,m);

D=zeros(m,1);

for i = 2:m

p1 = Fiducial_1(i,:);

p2 = Fiducial_2(i,:);

p3 = Fiducial_3(i,:);

p4 = Fiducial_4(i,1);

P_centroid_AFTRT = 1/3*( p1 + p2 +p3+p4);

q1 = p1 - P_centroid_AFTRT;

q2 = p2 - P_centroid_AFTRT;

q3 = p3 - P_centroid_AFTRT;

q4 = p4 - P_centroid_AFTRT;

H = q_1*q1 + q_2*q2 + q_3*q3 +q_4*q4;

[U,S,V] = svd( H ); % Singular value decomposition

X_temp = U*V.';

X = X_temp.';

d_temp = det(X);

if d_temp

R(:,:,i-1) = X;

end

D(i)=d_temp;

end

1. The stereotactic dose verification phantom (SDVP).

The Stereotactic Dose Verification Phantom (SDVP) provides dose measurements for commissioning treatment systems, such as Accuray CyberKnife, and specific plan dose verification.

The standard phantom is 20 x 20 x 10 cm in size, comprised of two 4 cm top and bottom build-up slabs, and two interchangeable 2 cm test inserts in the center. The Stereotactic Dose Verification Phantom is constructed of Blue Water material and additional slabs are available in many thicknesses for increased build-up. Gold and lead fiducial markers are located throughout the phantom for additional orientation and positioning accuracy. The side view and top view of stereotactic dose verification phantom is shown by Figure 1 and Figure 2.


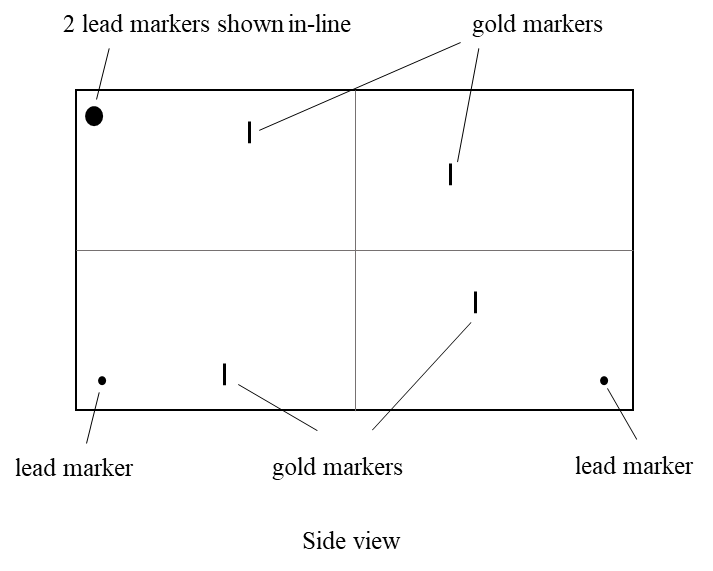


Figure 1. The side view of stereotactic dose verification phantom.


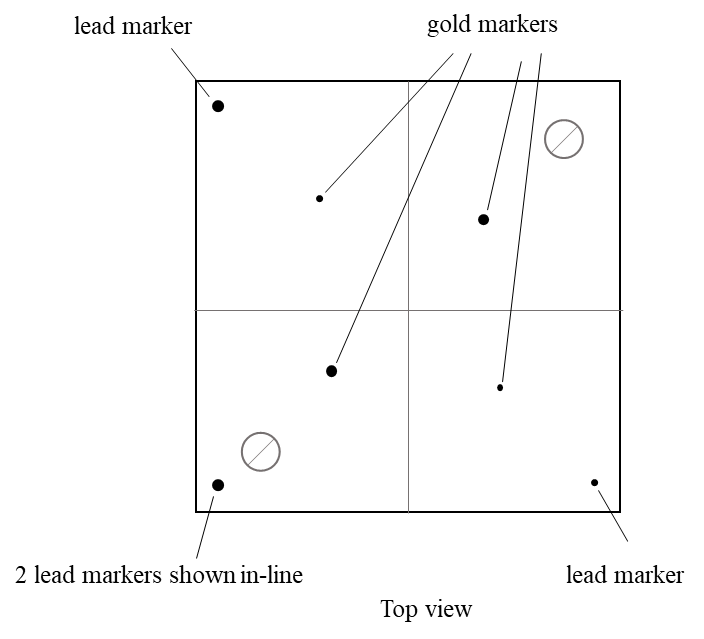


Figure 2. The top view of stereotactic dose verification phantom.
